# Validation of a Monte Carlo Framework for Out-of-Field Dose Calculations in Proton Therapy

**DOI:** 10.3389/fonc.2022.882489

**Published:** 2022-06-08

**Authors:** Marijke De Saint-Hubert, Nico Verbeek, Christian Bäumer, Johannes Esser, Jörg Wulff, Racell Nabha, Olivier Van Hoey, Jérémie Dabin, Florian Stuckmann, Fabiano Vasi, Stephan Radonic, Guillaume Boissonnat, Uwe Schneider, Miguel Rodriguez, Beate Timmermann, Isabelle Thierry-Chef, Lorenzo Brualla

**Affiliations:** ^1^ Research in Dosimetric Applications, Belgian Nuclear Research Center (SCK CEN), Mol, Belgium; ^2^ West German Proton Therapy Centre Essen WPE, Essen, Germany; ^3^ West German Cancer Center (WTZ), Essen, Germany; ^4^ Faculty of Medicine, University of Duisburg-Essen, Essen, Germany; ^5^ Radiation Oncology and Imaging, German Cancer Consortium DKTK, Heidelberg, Germany; ^6^ Department of Physics, TU Dortmund University, Dortmund, Germany; ^7^ Faculty of Mathematics and Science Institute of Physics and Medical Physics. Heinrich-Heine University, Düsseldorf, Germany; ^8^ Klinikum Fulda GAG, Universitätsmedizin Marburg, Fulda, Zurich, Germany; ^9^ Physik Institut, Universität Zürich, Zürich, Switzerland; ^10^ CEA, Université Paris-Saclay, Palaiseau, France; ^11^ Hospital Paitilla, Panama City, Panama; ^12^ Instituto de Investigaciones Cientificas y de Alta Tecnología INDICASAT-AIP, Panama City, Panama; ^13^ Department of Particle Therapy, University Hospital Essen, Essen, Germany; ^14^ Radiation Programme, Barcelona Institute for Global Health (ISGlobal), Barcelona, Spain; ^15^ University Pompeu Fabra, Barcelona, Spain; ^16^ CIBER Epidemiología y Salud Pública, Madrid, Spain

**Keywords:** proton therapy, anthropomorphic pediatric phantom, Monte Carlo simulation, out-of-field dosimetry, TLD, bubble detector

## Abstract

Proton therapy enables to deliver highly conformed dose distributions owing to the characteristic Bragg peak and the finite range of protons. However, during proton therapy, secondary neutrons are created, which can travel long distances and deposit dose in out-of-field volumes. This out-of-field absorbed dose needs to be considered for radiation-induced secondary cancers, which are particularly relevant in the case of pediatric treatments. Unfortunately, no method exists in clinics for the computation of the out-of-field dose distributions in proton therapy. To help overcome this limitation, a computational tool has been developed based on the Monte Carlo code TOPAS. The purpose of this work is to evaluate the accuracy of this tool in comparison to experimental data obtained from an anthropomorphic phantom irradiation. An anthropomorphic phantom of a 5-year-old child (ATOM, CIRS) was irradiated for a brain tumor treatment in an IBA Proteus Plus facility using a pencil beam dedicated nozzle. The treatment consisted of three pencil beam scanning fields employing a lucite range shifter. Proton energies ranged from 100 to 165 MeV. A median dose of 50.4 Gy(RBE) with 1.8 Gy(RBE) per fraction was prescribed to the initial planning target volume (PTV), which was located in the cerebellum. Thermoluminescent detectors (TLDs), namely, Li-7-enriched LiF : Mg, Ti (MTS-7) type, were used to detect gamma radiation, which is produced by nuclear reactions, and secondary as well as recoil protons created out-of-field by secondary neutrons. Li-6-enriched LiF : Mg,Cu,P (MCP-6) was combined with Li-7-enriched MCP-7 to measure thermal neutrons. TLDs were calibrated in Co-60 and reported on absorbed dose in water per target dose (μGy/Gy) as well as thermal neutron dose equivalent per target dose (μSv/Gy). Additionally, bubble detectors for personal neutron dosimetry (BD-PND) were used for measuring neutrons (>50 keV), which were calibrated in a Cf-252 neutron beam to report on neutron dose equivalent dose data. The Monte Carlo code TOPAS (version 3.6) was run using a phase-space file containing 10^10^ histories reaching an average standard statistical uncertainty of less than 0.2% (coverage factor *k* = 1) on all voxels scoring more than 50% of the maximum dose. The primary beam was modeled following a Fermi–Eyges description of the spot envelope fitted to measurements. For the Monte Carlo simulation, the chemical composition of the tissues represented in ATOM was employed. The dose was tallied as dose-to-water, and data were normalized to the target dose (physical dose) to report on absorbed doses per target dose (mSv/Gy) or neutron dose equivalent per target dose (μSv/Gy), while also an estimate of the total organ dose was provided for a target dose of 50.4 Gy(RBE). Out-of-field doses showed absorbed doses that were 5 to 6 orders of magnitude lower than the target dose. The discrepancy between TLD data and the corresponding scored values in the Monte Carlo calculations involving proton and gamma contributions was on average 18%. The comparison between the neutron equivalent doses between the Monte Carlo simulation and the measured neutron doses was on average 8%. Organ dose calculations revealed the highest dose for the thyroid, which was 120 mSv, while other organ doses ranged from 18 mSv in the lungs to 0.6 mSv in the testes. The proposed computational method for routine calculation of the out-of-the-field dose in proton therapy produces results that are compatible with the experimental data and allow to calculate out-of-field organ doses during proton therapy.

## 1 Introduction

Proton therapy (PT) enables to deliver highly conformed dose distributions owing to the characteristic Bragg peak and the finite range of protons. Nevertheless, PT is unavoidably accompanied by the production of secondary high-energy neutrons in the patient and structural materials of the beamline (1). Neutrons are of particular concern, as they are capable of traveling large distances to deposit out-of-field doses in organs located far from the primary treatment field and with a relatively high biological effectiveness (2). Furthermore, non-elastic nuclear reactions will also produce secondary protons, heavier ions, and gammas. As a result, the out-of-field radiation field in PT comprises a mixed field of radiation (including photons, neutrons, protons, and other charged particles) all with different potentials to induce biological damage. Moreover, the out-of-field radiation field, and hence the secondary dose delivered to healthy tissues, largely varies with position (close to field versus far) and depends on specific treatment parameters such as patient size and positioning, beam angles, proton energies, field size, modulation width, presence of range shifters (RSs), and the use of apertures.

The development of a validated Monte Carlo (MC) framework forms an important aspect in the assessment and characterization of out-of-field doses in PT. Nevertheless, the use of general-purpose MC simulations in out-of-field dosimetry is often restricted to detector calibration, and it has highlighted important differences between MC codes and models (3). Moreover, the coupling of MC to advanced measurement and proper benchmarking of the MC codes and models are still unknown today. Once validated, MC simulations will allow to fully describe the out-of-field radiation field and foster accurate calculations of appropriate dosimetric quantities needed for the assessment of radiation damage and risks.

Out-of-field dosimetry is especially important for the radiation protection of children who might develop radiation-induced second primary tumors during their lifetime. Nowadays, the challenge for clinicians is to increase the survival rate while treating with fewer secondary effects. There is a critical need to understand the long-term health and quality of life (QoL) challenges in these populations and to assess the potential health effects of the treatment modalities to improve the survival and health of the patients.

Some medical physicists are cautious that the existing knowledge and understanding of the out-of-field doses and associated risk of inducing secondary malignant neoplasms (SMNs) is not sufficiently mature to justify the use of modern techniques, such as PT, for treating children or pregnant women (4). Therefore, a full characterization of the out-of-field doses, particularly at the PT field edge, requires special attention for the radiation protection and prevention of SMNs (5).

This study aims to set up and optimize a computational MC framework for out-of-field dosimetry in PT, through validation measurements with advanced dosimetry techniques. The study herein presented has been conducted in the framework of a European Horizon 2020 project, HARMONIC, which is addressed at improving the knowledge of the health effects of medical exposure during childhood. A central task within HARMONIC is to set up a cohort of pediatric patients treated with modern radiotherapy, including the computation of whole-body doses. Ultimately, the goal is to estimate the risk of late health effects (including the risk of second primary cancers) after pediatric radiotherapy exposures, which relies on organ dose estimation obtained from validated tools.

## 2 Material and Methods

### 2.1 Experimental Setup

**Figure 1:**
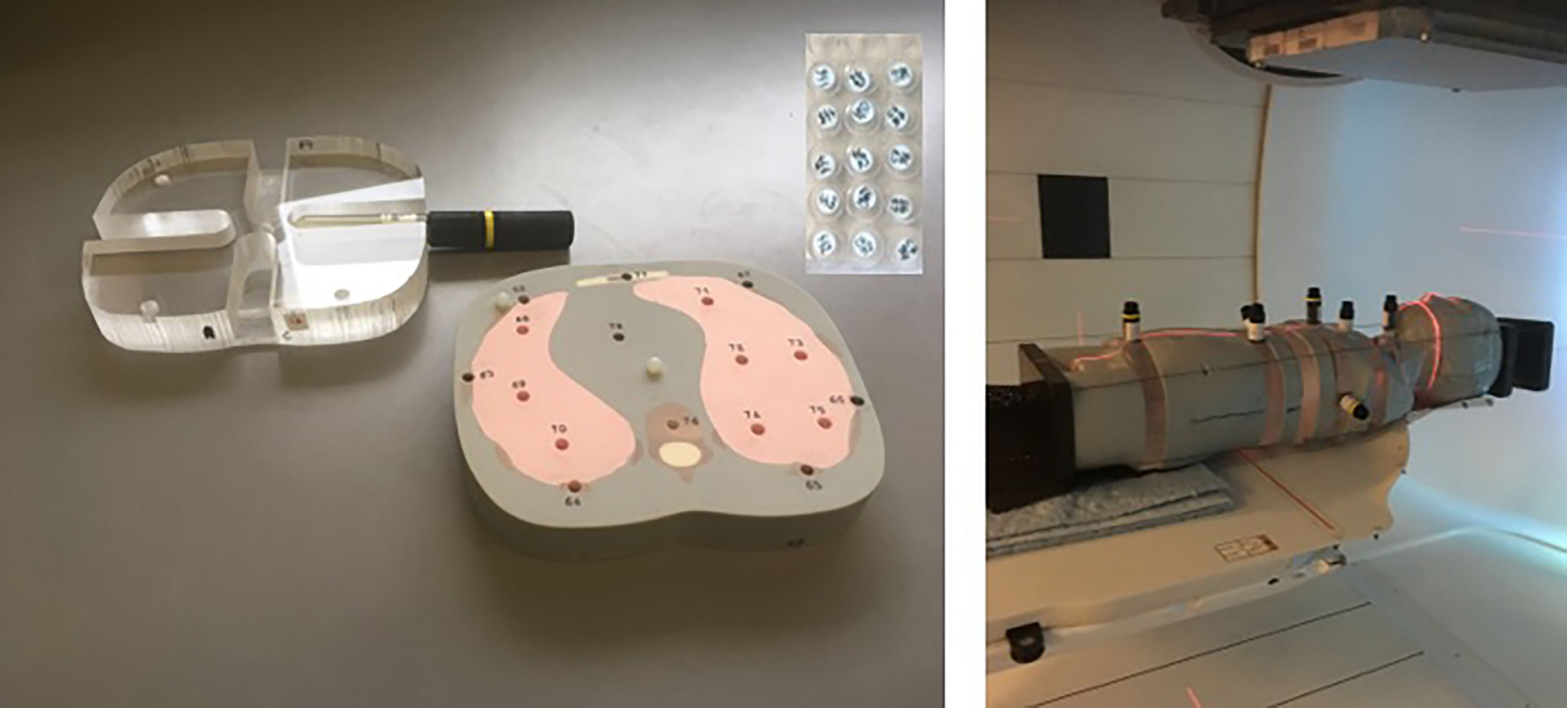
Pictures of the experimental setup. On the left are the slabs of the anthropomorphic phantom for insertion of TLDs including one of the designed PMMA slabs for BD-PNDs. On the right is the mounted 5-year-old anthropomorphic phantom (loaded with BD-PNDs) positioned in the gantry room for PT pencil beam scanning. TLDs, thermoluminescent detectors; PMMA, polymethyl methacrylate; BD-PNDs, bubble detectors for personal neutron dosimetry; PT, proton therapy.

For this study, an anthropomorphic phantom (ATOM, Computerized Imaging Reference Systems (CIRS), Inc., Norfolk, VA, USA) representing a 5-year-old child (type 705D) was used. The phantom consists of tissue equivalent (TE) materials, and 180 dosimeters can be inserted in different organ positions. For the insertion of bubble detectors, six tissue slabs were replaced by polymethyl methacrylate (PMMA) slabs manufactured at SCK CEN with dedicated inserts for this type of detector (see **Figure 1**).

Aiming to simulate a realistic proton treatment of a brain tumor, a clinically applied treatment plan was transferred to the conditions of the experiment. The corresponding patient should feature a cranial size and shape with a reasonable resemblance to the corresponding features of the anthropomorphic phantom. A 7-year-old female patient was selected with a diffuse midline glioma (WHO grade IV). The patient received combined radiotherapy and chemotherapy after R3 resection. A median dose of 50.4 Gy(RBE) with 1.8 Gy(RBE) per fraction was prescribed to the initial planning target volume (PTV), which was located in the cerebellum and had a volume of 195.2 cm^3^. The treatment plan consisted of two ipsilateral oblique fields and a contralateral oblique field. The proton fields were delivered in a gantry room in pencil beam scanning (PBS) delivery mode employing a lucite RS with a physical thickness of 4.44 cm and a water-equivalent thickness of 5.14 cm. The concerned patient was enrolled in the prospective registry study “KiProReg” (German Clinical Trials Register: DRKS-ID: DRKS00005363) after consent from her legal guardians was obtained. This study was approved by the local ethics committee.

The treatment planning of the phantom case was conducted in the treatment planning system (TPS) RayStation (version 7, RaySearch Laboratories, Stockholm, Sweden). The X-ray CT image set was contoured in the following way: the boundaries between dissimilar TE material types were delineated; then the mass density of the volumes of the individual TE materials was overwritten with the values from the datasheet of the phantom. Furthermore, the volumes containing the thermoluminescent detector (TLD) inserts were contoured. A deformable image registration (6) was established between the clinical CT and the anthropomorphic phantom. Then the contours of the target volume and organs at risk (OARs) were mapped to the CT of the phantom. After that, the contour of the PTV was fine-tuned by alignment to the boundaries between brain substitute tissue and bone substitute tissue. The proton kinetic energies of the fields ranged from 100 to 165 MeV. The air gap between the RS and phantom surface was on average 11.0 cm regarding the minimum distance and an average of 14.6 cm regarding the distance on the central axis. The dose distribution was calculated with the MC-based dose engine (version 4.1) of RayStation. The objectives of the spot fluence optimization were similar to those of the clinical plan concerning, i.e., the right cochlea, the brainstem, and the PTV.

The delivered dose in the experiments was adjusted to the sensitivity and location of the corresponding detectors. The phantom irradiation plans for TLD measurements applied 100.8 Gy(RBE) to the PTV in a single fraction. For bubble detectors, the delivered dose to the PTV ranged between 0.5 Gy(RBE) for close-to-field measurements and 6 Gy(RBE) for far out-of-field positions. Large changes in the dose level by modification of the overall number of monitor units (MU) are impossible in PBS because the allowed MU per beamlet (“spot”) is subject to machine limitations. Thus, dedicated optimizations of the spot fluence were conducted per targeted dose level. This also included an adaptation of the spot spacing. As a result, the shape of the corresponding dose distributions was not exactly identical. This concerns, however, only the high dose region, which was not the subject of the current study.

The experiments were conducted in the West German Proton Therapy Centre Essen (WPE), which is based on the ProteusPlus proton machine (IBA PT, Louvain-La-Neuve, Belgium). Protons were accelerated in an isochronous cyclotron and subsequently slowed down in the energy selection system to adapt to the required range in the phantom. The protons were guided to a gantry-mounted, evacuated nozzle, which operated in a spot-by-spot type PBS delivery mode. The RS was mounted in a snout holder, which could be moved along the central beam axis with a linear translation stage. The cranial part of the phantom was put on a BoS Headframe (Qfix, Avondale, PA, USA), which in turn was attached to a short Patlog table (IBA PT, Schwarzenbruck, Germany). The phantom was aligned prior to the mock treatment with the lasers of the positioning system. The uncertainty was about ±2 mm. Although it could have been reduced with the X-ray-based verification system, this was not done to avoid a contribution of X-rays to the detector signal.

### 2.2 Dosimetry Systems

#### 2.2.1 Thermoluminescent Detectors

TLDs, produced by IFJ-PAN (Krakow, Poland), are small cylindrical chips with a diameter of 4.5 mm and a height of 0.9 mm. Detectors of Li-7-enriched LiF : Mg, Ti (MTS-7) type were used. During PBS therapy, MTS-7 mainly detects gamma radiation, which is produced by nuclear reactions, and primary or secondary as well as recoil protons created out-of-field by neutrons. MTS-7 sensitivity to neutrons is very limited. However, Li-6-enriched LiF : Mg,Cu,P (MCP-6) detectors are very sensitive to thermal neutrons due to their high ^6^Li(n,*α*)^3^H cross section for thermal neutrons. MCP-6 was used in combination with Li-7-enriched MCP-7 to quantify thermal neutrons.

TLD detectors were read in a Thermo Scientific Harshaw 5500 reader following a preheat for 30 min at 120°C to avoid the effects of signal fading and low-temperature anomalies in the glow curves (Parisi 2018). A heating rate of 10°C/s was used to heat up TLDs up to 340°C for MTS type and up to 255°C for MCP type.

TLDs were calibrated with a Co-60 source in terms of kerma “free in air” (*K*_air_), which was then converted to absorbed dose to water (*D*_W_) using conversion factor *D*_W_/*K*_air_ = 1.12 determined by the ratio of the mass energy absorption coefficient for water to air for the energy of Co-60 (7). MTS-7 data were expressed in absorbed dose in water per target dose (physical) [μGy/Gy]. To quantify the thermal neutron dose, the data from MCP-7 were subtracted from MCP-6. Next, we applied *K*_air_ to neutron dose equivalent conversion coefficients for thermal neutrons (1.24 * 10^−02^ mSv/mGy), as described in (8). However, it should be noted that this conversion coefficient has been reported to have an uncertainty of up to a factor 2, which is related to the uncertainties on energy and angular distribution of neutrons as well as on the light collection of the TLD reader (8). Finally, data were expressed as thermal neutron dose equivalent data (μSv), which were normalized to the physical target dose (Gy) and expressed as [μSv/Gy]. The estimated uncertainty was 100% (8).

Uncertainties on the out-of-field absorbed dose were assessed considering dosimeter reproducibility (1.8%), batch reproducibility (1.9%), Co-60 calibration uncertainty (2.4%) (9), and background uncertainties, which were dependent on the measured dose and reached up to 11% (coverage factor *k* = 1) for the farthest positions. For the energy response of MTS-7 detectors, the energy dependence for both photons (10) and protons (11) was considered. For the energy dependence of photons, a characteristic spectrum was used in the study of (12). Photon energies ranged between 30 keV and 10 MeV. Assuming a flat energy dependence above 1 MeV (10, 13), the calculated uncertainty was below 1% (*k* = 1). For the proton energy dependence, a uniform distribution over the proton energies was assumed for energies up to the maximum proton energy used in this study (165 MeV). This resulted in an uncertainty on the proton energy response of 5% (*k* = 1).

#### 2.2.2 Bubble Detectors

Bubble detectors for personal neutron dosimetry (BD-PNDs) (Bubble Technology Industries, BTI, Chalk River, ON, Canada) were used to measure neutrons of energies above 50 keV. These cylindrical detectors are 15 cm in length and 2 cm in diameter, but the sensitive part, where bubbles are created, is only 7 cm in length and 1.6 cm in diameter (see **Figure 1**). BD-PNDs were calibrated with a Cf-252 source to obtain neutron dose equivalent by applying fluence-to-dose equivalent conversion factors derived from kerma factors *k*(*E*) and a quality factor as a function of neutron energy (*Q*(E)) for ICRU tissue, as described previously (14). The final data were expressed in neutron dose equivalent per target dose (physical) [μSv/Gy]. Uncertainties of BD-PNDs are estimated to be on average 20% (*k* = 1).

### 2.3 Monte Carlo Framework

The well-established Geant4 (15–17) wrap-up MC code TOPAS v3.6 (Geant4) (18), in conjunction with the Matlab (The Mathworks, Inc., Natick, MA, USA)-based matRad v2.10.1 (19) project to create a DICOM-based dose verification system, was used to simulate the out-of-field absorbed dose distribution. For this purpose, matRad was extended by including the possibility to process DICOM RTIon files. With this feature, it was then possible to create the TOPAS input files with the treatment room-specific radiation parameters employing matRad as the TOPAS syntax parser. The simulations for the determination of the neutron equivalent dose at a point and the proton and gamma out-of-field dose could then be conducted.

#### 2.3.1 Beam Model

To simulate the anthropomorphic phantom irradiation, it was necessary to run TOPAS simulations that reproduce the commissioned beam. Mean energy and spread have been adjusted to reproduce the measured depth dose curves following the methods of (20, 21), and (22) in 5 MeV steps from the lowest energy available (that is, 100.0 MeV) up to the highest energy available (that is, 226.7 MeV). The commissioned beam data for the corresponding reference values were yielded by measurements with the plane parallel Bragg peak chamber (PTW, Freiburg, Germany) (23). Simulated and measured depth doses agreed within ±0.01 cm at *R*_80_. In addition, the Fermi–Eyges parameters from the beam model implemented in RayStation were used to fully characterize the proton pencil beam.

Furthermore, an MU/ion calibration was performed to determine the number of protons in TOPAS corresponding to the respective MUs. For this purpose, reference fields, consisting of 1,681 spots with 0.25-cm spacing arranged in a symmetrical square around the isocenter, were simulated with 5 × 10^5^ protons per spot for the 27 different energies that make up the beam parameter database. The protons started 50 cm upstream of the isocenter at the nozzle exit of the treatment head. The Fermi–Eyges parameters were back-projected in vacuum to the nozzle exit (24–26). The method of (27) was applied to obtain the spot positions of the protons, taking into account the deflection of the protons at the two foci from the scanning magnets of the pencil beam dedicated nozzle. Downstream, a water tank with a volume of 50 × 50 × 50 cm^3^ was created. The isocenter was at 3-cm depth of the water tank. Simulation conditions thereby correspond to the conditions for beam-monitor calibration at the WPE, generally following reference dosimetry according to TRS 398 and DIN 6801-1 (28, 29). In the simulations, a cylindrical tally with a diameter of 1 cm and a thickness of 0.5 cm was then placed at the isocenter. The following physics models were employed (30): *g4em-standard_opt4*, *g4h-phy_QGSP_BIC_HP*, *g4decay*, *g4ion-binarycascade*, *g4h-elastic_HP*, and *g4stopping*. In accordance with (31), the mean excitation energy of G4 water was set to 78 eV. The density was set to 1 g/cm^3^. The overall allowed maximum step size for the condensed history algorithm in TOPAS was set to 0.1 cm. The production cut for all secondary particles was set to 0.05 cm. No variance-reduction techniques were employed. These simulations reached a standard statistical uncertainty of less than 0.6%.

The MU/ion calibration was then performed employing the simulation result of the tally in dose [Gy/MU] and using the dose meter set in [Gy(RBE)/MU]. The calibration yielded was then added to the beam parameter database so that the treatment room-specific machine parameter file included 27 mean energies, energy spreads, Fermi–Eyges parameters, and the number of protons per single MU.

#### 2.3.2 RT Integration in TOPAS

The matRad code was extended to read DICOM RTIon files including both RT Plan and RT Struct. The RS used was considered in the matRad configuration files with the 4.44-cm-thick material lucite comprising a mean excitation energy of 74.0 eV, a density of 1.19 g/cm^3^, and material composition of 8.05% H, 59.98% C, and 31.96% O, corresponding to the material definition as included in the PSTAR database given by the National Institute of Standards and Technology (NIST). Since RayStation specifies spot positions at the isocenter, these were back-calculated analogously to the procedure for MU/ion calibration. Based on the machine parameter file, the required beam data were linearly interpolated starting from the energies given by the RayStation RT Plan file for the individual energy layers. The DICOM CT images were incorporated into TOPAS using the *TSImageCube* function. The device and scan protocol-specific density corrections were applied, as well as the full Schneider model comprising 25 different stoichiometric tissues (32). The grid size of the inserted CT was in accordance with the one employed in RayStation, that is, 0.2 × 0.2 × 0.2 cm^3^.

The matRad and TOPAS build was validated with a simulation in which the absorbed dose to water was tallied and compared with the RayStation simulation by means of a 3D gamma test. The 10^9^ simulated histories were distributed based on the respective MU weights per spot. The simulations were run on four Intel^®^ Xeon^®^ CPU E5-2670 v3 @ 2.30GHz (48 cores) with 64.0 GB RAM each. The three applied fields were divided into four runs each so that a total of 12 individual simulations were necessary to obtain the full dose distribution. The number of histories relative to the distribution of MUs for each spot was sufficient to avoid undersampling by more than 0.01%. The results from the simulations were then obtained by summing the individual runs of the respective fields. Since the number of histories corresponding to the median dose of 50.4 Gy(RBE) is known, it can be multiplied by the appropriate factor to obtain a comparable dose within the matRad-based analysis of the results.

#### 2.3.3 Out-of-Field Dose Calculations

According to (33) and (34), for the simulation of the neutron equivalent dose at a point and the gamma dose, respectively, the geometry of the treatment room and the gantry pit must be implemented in the MC code in order to obtain a comprehensive neutron spectrum including thermal neutrons and a gamma dose to water as accurately as possible. A fully rotating gantry around the isocenter of the treatment room was modeled in TOPAS. The geometrical models of the scanning and bending magnets, as well as of the counterweight, were simplified. The geometry also included a rotating table, a maze, a rolling floor, and a gantry pit including all walls, ceilings, and floor with the corresponding materials of the treatment room and gantry pit. The simulation environment thus had a volume of 20.0 × 7.0 × 20.0 m^3^. The world material in these simulations was altered from vacuum to air to account for ionization occurring in the air relevant for thermal neutrons and gammas. The physical properties of air were modeled according to (31). However, to ensure that the initial protons hit the upstream side of the RS as accurately as possible with respect to the Fermi–Eyges parameters, the initial proton transport started in a vacuum box with the lateral dimensions of the RS, and a longitudinal extension of 50 cm upstream of the isocenter to the upstream side of the RS. The additional energy loss due to air scattering downstream the RS, up to the surface of the anthropomorphic phantom, was accounted for *via* a slight increase in the mean energy of each spot. This increase was calculated by an interpolation employing the data provided in the PSTAR database for the continuous slowing down approximation in air based on a logarithmic cubic spline fit and the predicted track length of the protons. The influence of the additional air scattering on the energy spread is negligible, as well as the number of initial protons absorbed in air subsequent to the interaction in the RS.

The out-of-field dose simulations used 10^10^ primary protons. The simulation parameters were set analogously to those described above for the MU/ion calibration. The gamma and proton dose to water, as well as the neutron dose equivalent at a point, were tallied. To determine the neutron dose equivalent at a point, fluence-to-dose conversion factors within the TOPAS *AmbientDoseEquivalent* scorer were employed based on a logarithmic energy binning. The built-in conversion factors were adopted to the appropriate fluence-to-neutron dose equivalent conversion factors as described in the study from (14) by using tissue kerma factors *k*(*E*) and quality factors as a function of neutron energy (*Q*(*E*)). In addition, simulations under identical conditions were performed in which only the dose to water of secondary protons was scored to elaborate on the effect of high-energy protons close to the irradiation field.

The spatial grid size in the TOPAS simulations was chosen analogously to the grid size in the RayStation simulations, namely, 0.2 × 0.2 × 0.2 cm^3^. The doses for the neutron dose equivalent at a point, as well as the gamma and proton dose to water, were obtained from reading out the RTStructs of the contoured TLD positions *via* averaging of the voxels comprising the respective areas.

### 2.4 Calculation of Total Dose Equivalent and Organ Doses

Following validation of the MC framework, TOPAS simulations were used to calculate the total dose equivalent. The contribution of the following particles was considered for the out-of-field dose, and their contributions were added to obtain the total dose equivalent:

1. Primary protons and assuming a generic RBE of 1.1.
2. Secondary gammas considering an RBE=1.
3. Neutrons as calculated according to the method described in Section 2.3.3.

Finally, organ doses were calculated relying on the specific locations of TLD inserts in the CIRS phantom, which correspond to certain organs to allow for organ dosimetry. In a total of 28 organ doses per target, the dose was calculated as well as the total dose to the child’s organs considering a total target dose of 45.8 Gy (physical dose), i.e., 50.4 Gy(RBE).

## 3 Results

### 3.1 Measured Out-of-Field Doses

#### 3.1.1 MTS-7 Thermoluminescent Detectors

**Figure 2:**
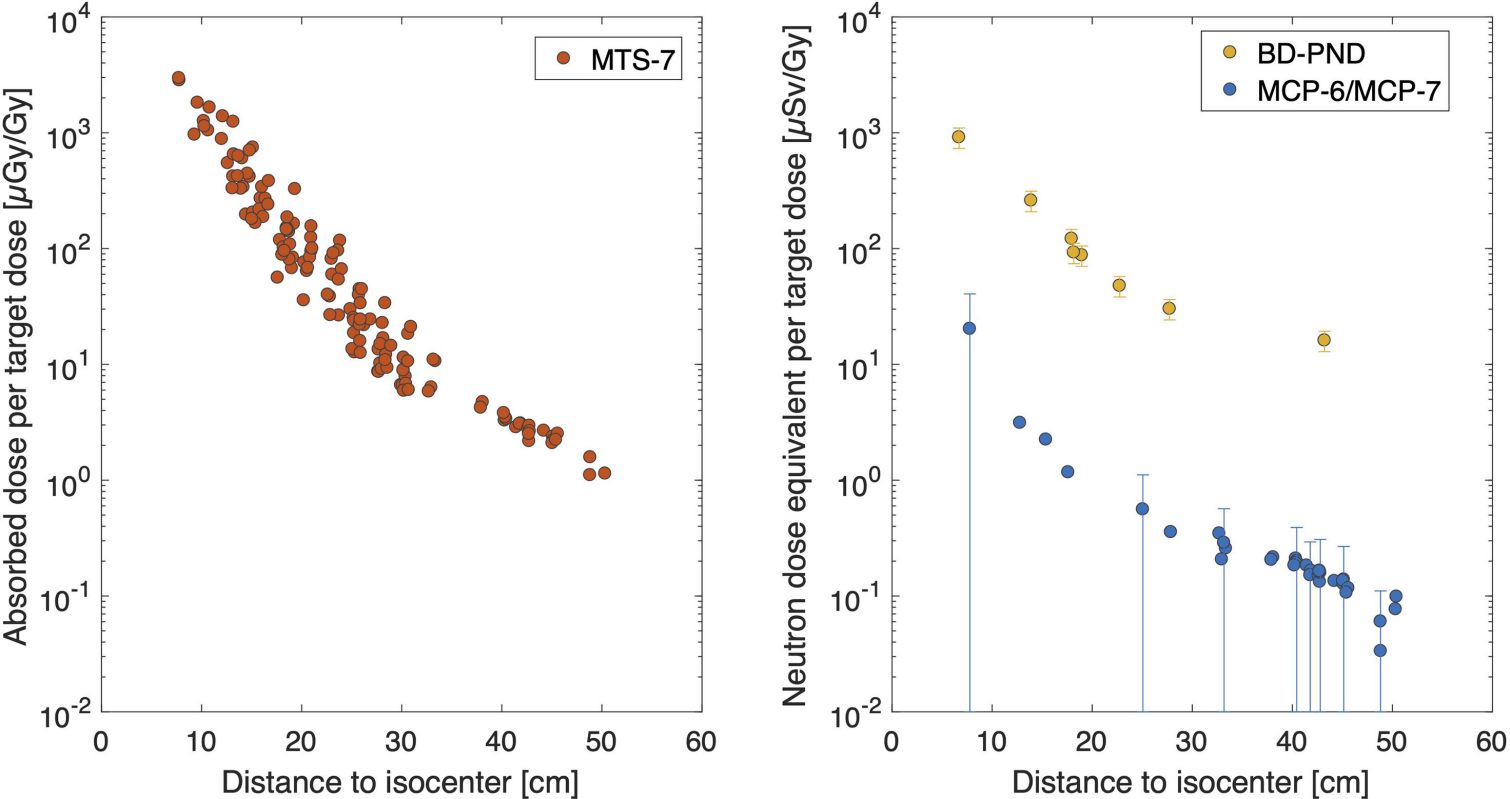
Overview of experimental data. The absorbed dose in water per target dose [μGy/Gy] is plotted as a function of distance for MTS-7 (left figure). The uncertainty bars in this figure are not displayed since they are smaller than the symbol size. In the right figure, the neutron dose equivalent data are plotted as a function of distance for BD-PND and MCP-6 in combination with MCP-7. Uncertainty bars (*k* = 1) are plotted for all BD-PNDs and for MCP-6/MCP-7 for every 5th data point to maintain readability of the plot. Notice that the abscissas and ordinates axes of both figures are the same for comparison purposes.

Absorbed dose in water per target dose, as measured with MTS-7 detectors, revealed doses ranging from 2,842 ± 181 to 7.9 ± 0.5 μGy/Gy at respectively 7.8 and 50 cm from the isocenter (see **Figure 2**). Compared to the target dose, the out-of-field dose was more than 2 orders of magnitude lower close to the field and decreased to 5 orders of magnitude lower doses beyond 35 cm out-of-field. MTS-7 results report on the absorbed dose in water from non-neutron contributions, which are dominated by protons and also gammas that contribute to their signal. Close to the field, one can expect a larger contribution of primary protons, while further away from the field, these primary protons will not be measured, as recoil protons will dominate the field.

#### 3.1.2 MCP-6 and MCP-7 Thermoluminescent Detectors

Thermal neutron dose was obtained by subtracting MCP-7 from MCP-6 detectors. The neutron dose equivalent per target dose ranged between 20.3 and 0.08 μSv/Gy for 7.7 to 50 cm from the isocenter, respectively (see **Figure 2**). It should be noted that the uncertainty (*k* = 1) on the calibration factor is 100%, which is due to a number of different contributions as described in (8). These data can also be expressed in gamma equivalent neutron doses (not plotted), as it is sometimes referred to by other groups to quantify the thermal neutron dose in terms of gamma dose equivalent. This easy approach is obtained by MCP-6 minus MPC-7 doses as calibrated in Co-60 dose in water (*D*_W_). Hence, gamma equivalent neutron doses in this study ranged between 1846.0 and 6.9 μGy/Gy.

#### 3.1.3 Bubble Detector for Personal Neutron Dosimetry

Finally, it was possible to obtain the neutron dose equivalent (neutron energies greater than 50 keV) in 6 different positions. Bubble detector data doses were between 915 ± 183 and 16 ± 3 μSv/Gy for respectively 6.7 cm and 43 cm from the isocenter (see **Figure 2**). When comparing these results with the data obtained by MCP-6 in combination with MCP-7, as described in Section 3.1.2, it was noted that the thermal neutron dose equivalent data were much lower compared to the neutron dose equivalent measured by BD-PNDs (neutron energies greater than 50 keV). More specifically, the thermal neutron dose contribution to the total neutron dose was only 2.5% close to the field and decreased to 0.9% at 50 cm.

### 3.2 TOPAS Simulations

#### 3.2.1 Target Dose Distribution

**Figure 3:**
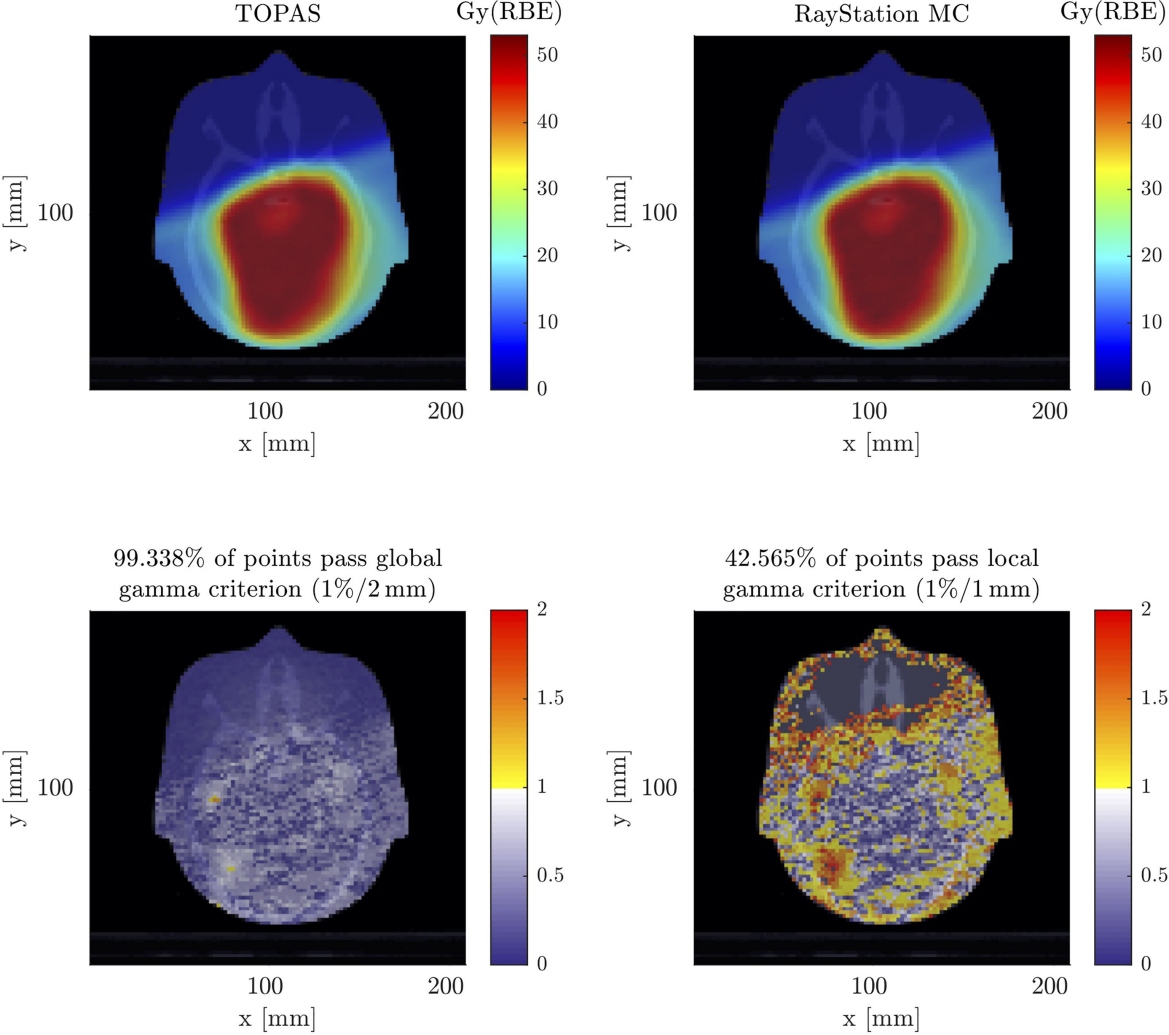
Comparison between the absorbed dose distribution calculated by TOPAS (top left) and the absorbed dose distribution calculated by RayStation (top right), both shown in axial plane. A 3D gamma test was performed once for a global gamma of 1%, 2 mm (bottom left), and for a local gamma of 1%, 1 mm (bottom right), both excluding the dose outside the anthropomorphic phantom so as not to bias the test result.

**Figure 3** shows the result of the simulated absolute absorbed dose distribution from TOPAS. The dose to water was scored in order to compare to the RayStation results employing a 3D gamma test. On the lower left side in **Figure 3**, it can be seen that for a global 3D gamma test with passing criteria of 1%, 2 mm produces a gamma pass rate of 99.338%. This makes it evident that the implementation of the verification system was successful. To ensure that the differences between the overwritten materials of the anthropomorphic phantom in RayStation and the Schneider model did not cause discrepancies, the *R*_80_ range of each simulated irradiation field from TOPAS was analyzed with the corresponding *R*_80_ ranges of the fields from RayStation, where discrepancies were smaller than ±0.02 cm.

On the lower right side in **Figure 3**, it can be seen that in a local 3D gamma test with 1%, 1 mm, the obtained gamma pass rate was 42.565%. It is recognized that most of the deviations occur outside the prescription range in this sensitive gamma test.

#### 3.2.2 Out-of-Field Dose Simulations

**Figure 4:**
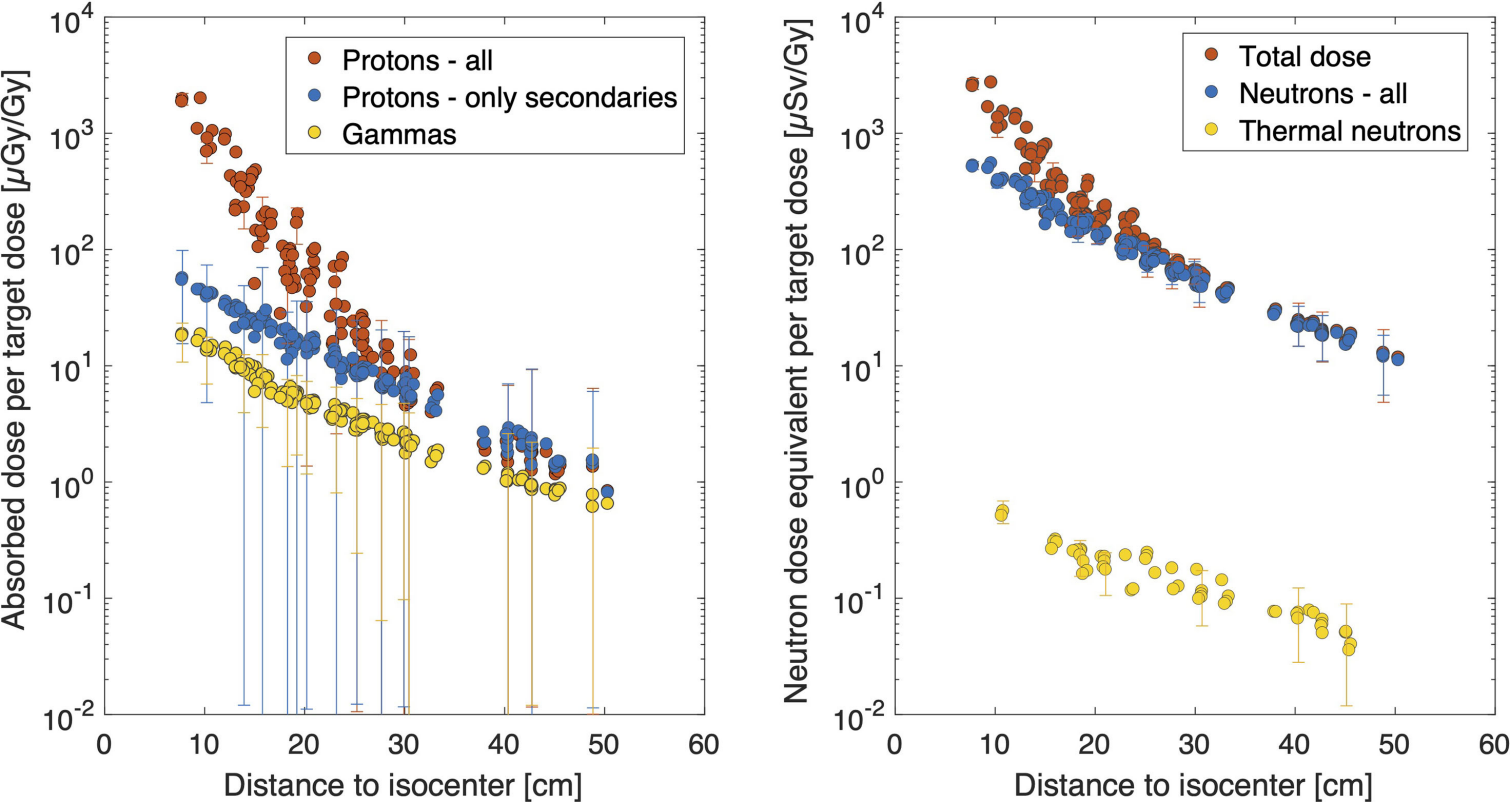
Overview of TOPAS results. Absorbed dose in water per target dose [μGy/Gy] is calculated for all protons, only secondary protons and gammas (left figure). Neutron dose equivalent data are plotted as a function of distance for all neutron energies considered, and when only considering the thermal neutrons (right figure). Uncertainty bars (k = 1) are plotted only once every 10th data point to maintain readability of the plot.

In the top part of **Figure 4**, results demonstrate absorbed doses from different contributors including protons, all protons as well as secondary protons and gammas. Results show that the proton dose was approximately 2 mGy/Gy close to the field and drops to 1 μGy/Gy, at the very end of the phantom. Looking into the secondary protons, they match the total proton dose from 30 cm and higher, which points toward the fact that at only beyond 30 cm, primary protons will not contribute to the out-of-field doses. Secondary protons can be generated from the primary beam (which will be absorbed close to the field), but most likely they are created by neutrons as recoil protons. The gamma-induced dose was always lower than the proton dose and ranged on average between 1% and 54% of the proton dose close to the field and far out-of-field (50 cm), respectively. On the contrary, when neglecting the primary protons and only considering secondary protons, the gamma dose was on average 30% and 70% close to the field and far out-of-field, respectively. It should be noted that the uncertainty bars become wider the further the dose has been simulated out-of-field. This is related to the decreased particles in these regions hitting the small TLD volumes. To avoid biasing the data, no variance reduction techniques have been applied in the simulations.

Neutron dose equivalent considering all neutrons and only thermal neutrons are shown in the right plot in **Figure 4**. Data demonstrate a neutron dose equivalent of 528 ± 41 μSv/Gy at a 7.8-cm distance, which decreases to 11.2 μSv/Gy at 50 cm from the isocenter. Clearly, the contribution from thermal neutrons to the total is very small and remained below 1%. Assuming the secondary proton production is mainly from recoil protons generated from neutrons, we also calculated the average quality factor from these data. We divided the calculated neutron dose equivalent data by the absorbed dose quantity from secondary protons and derived an average Q-factor of approximately 10, which did not vary significantly between locations out-of-field and is in line with literature data. Finally, the left plot in **Figure 4** also shows the total dose equivalent per target dose, which considers the contribution of the primary protons close to the field edge. At larger distances, it is clear that the total dose equivalent is dominated by the neutron dose, as the gamma dose does not contribute significantly to the dose equivalent (1%–6%).

### 3.3 Comparison of Out-of-Field Doses

**Figure 5:**
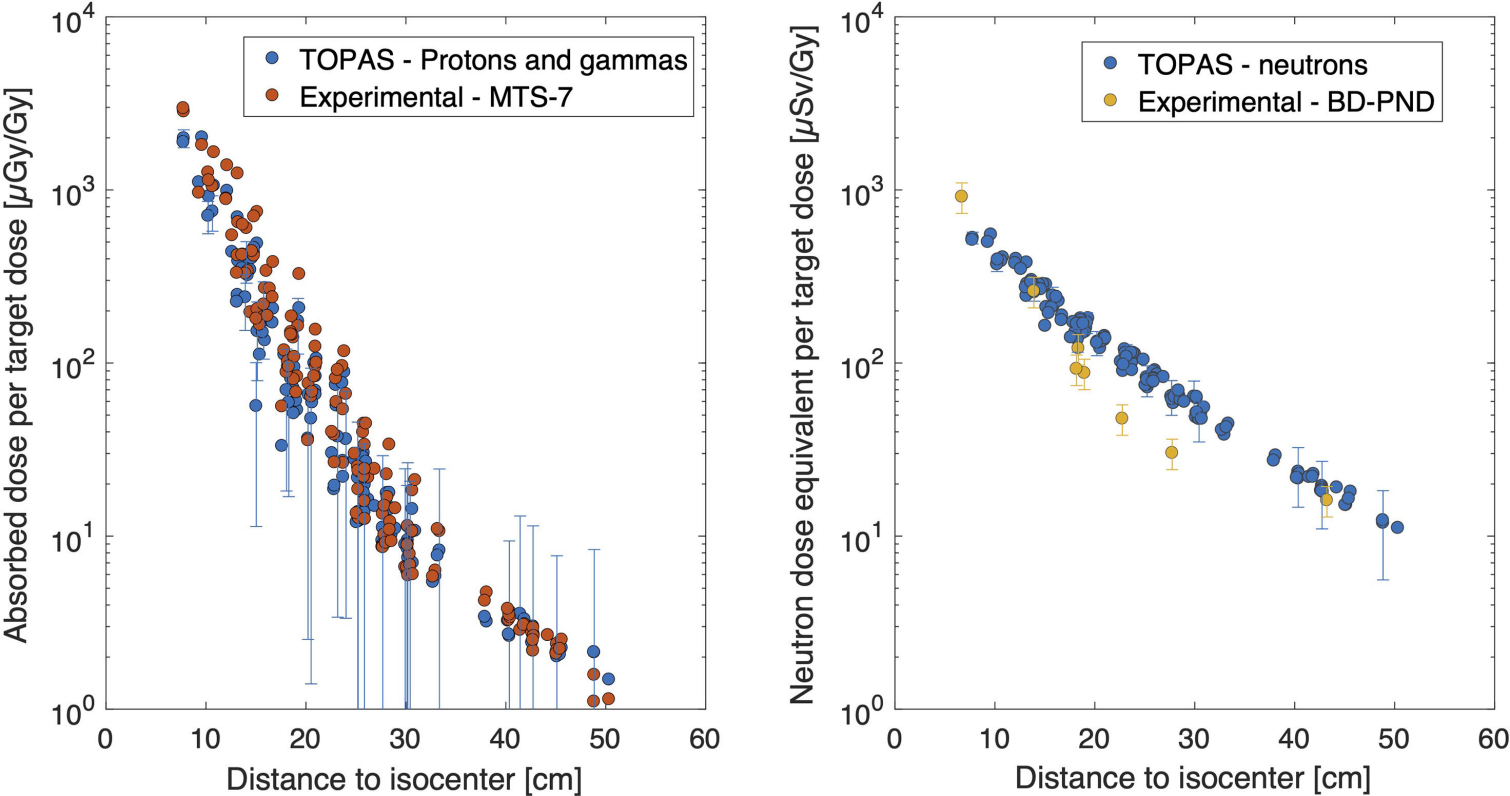
Comparison between experimental data and TOPAS. On the left, the absorbed dose in water per target dose [μGy/Gy] is plotted for MTS-7 measurements and TOPAS simulations summing proton and gamma doses. On the right, neutron dose equivalent data are plotted for BD-PND and TOPAS simulations of neutron doses. BD-PND, bubble detector for personal neutron dosimetry.

In **Figure 5**, a comparison between the dose obtained from the MTS-7 detectors and TOPAS, from both protons and gamma contributions, revealed a good agreement. On average, the TOPAS doses, including proton and photon contributions, were 18% lower compared to those from the MTS-7 detectors with a slightly better agreement at larger distances. Nevertheless, the last 3 data points reveal a lower experimental dose compared to the TOPAS dose, which was within uncertainties, due to the larger uncertainties of the calculations at these positions as well as the higher uncertainties of the measurement points, as measured doses are closer to the background, and background uncertainties are 11%. In the right plot of **Figure 5**, the comparison between the experimental data obtained with BD-PNDs and simulated neutron dose equivalent data shows a good agreement close to the field and far from the field. Nevertheless, at 23 and 28 cm, the measured data were 50% lower compared to the simulated ones. One should keep in mind the use of PMMA slabs for the insertion of bubble detectors, which may have an impact on the out-of-field doses, particularly in regions where the density of the phantom material should be lower such as the lung region. Moreover, bubble detectors have an uncertainty of 20% (*k* = 1), which does not include the uncertainty related to their energy response, as this is not known for energies above 20 MeV.

**Figure 6:**
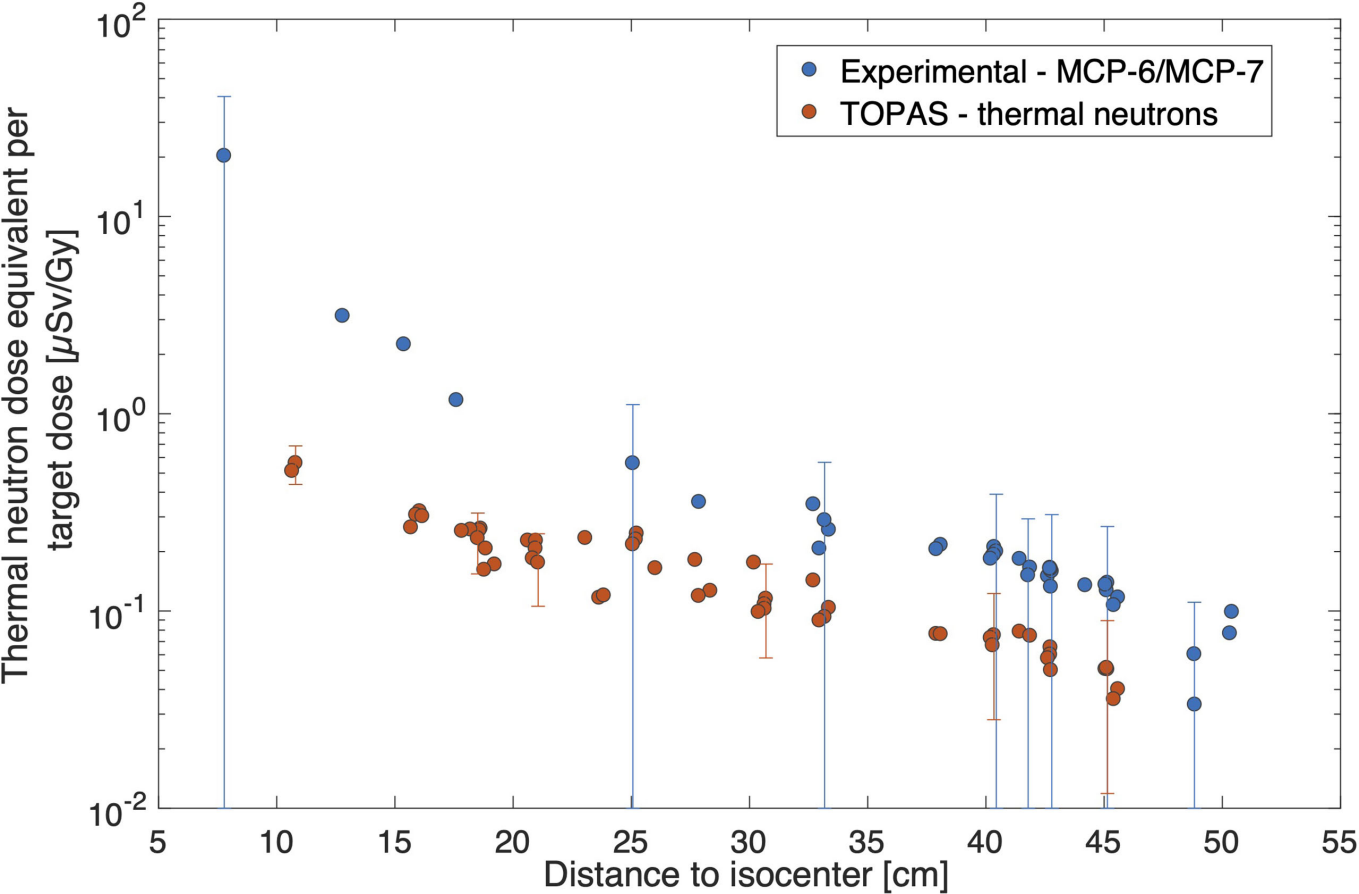
Comparison of thermal neutron doses per target dose [μSv/Gy] for experimental data from MCP-6/MCP-7 data and TOPAS simulations. Uncertainty bars (*k* = 1) are plotted once every five points for clarity reasons.

The measured thermal neutron doses are plotted in **Figure 6** together with the MC calculated thermal neutron doses. The experimental doses are higher than the simulated data. The difference is the largest close to the field where a 15-fold higher dose was measured, while out-of-field, the experimental data were a factor of 2 higher but within uncertainties.

### 3.4 Organ Dose Calculations

**Figure 7:**
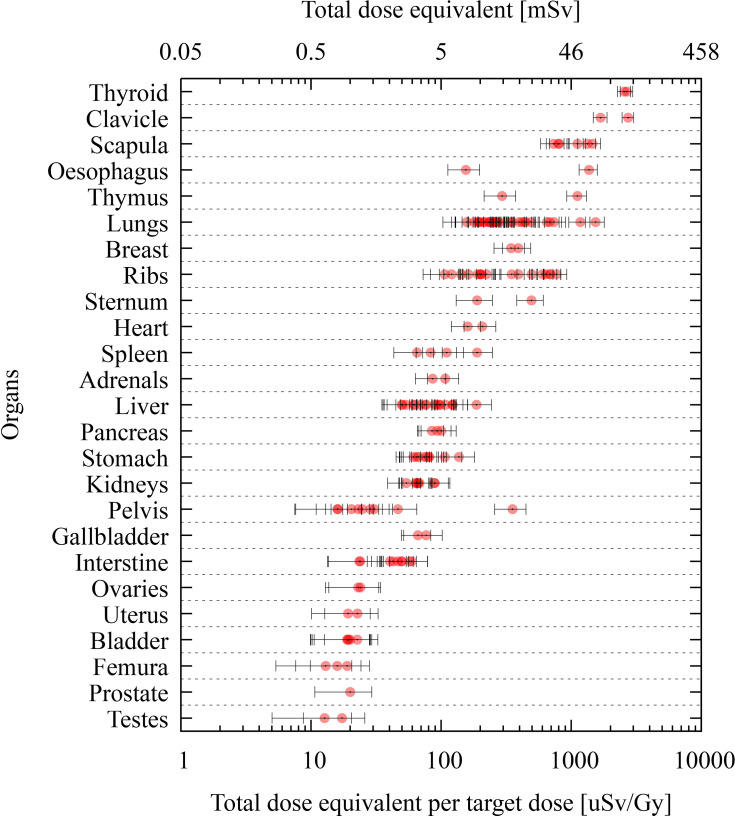
Organ dose calculations produced by TOPAS at various TLD positions of the phantom. Data are grouped per organ, and the dose is reported as total dose equivalent per target dose [μSv/Gy] in lower abscissas and total dose equivalent for a target dose of 45.8 Gy in the upper abscissas. Uncertainty bars (*k* = 1) are given for each position.

Dose calculations were grouped per organ in **Figure 7**, using a total target dose of 45.8 Gy (50.4 Gy(RBE)). The average thyroid dose per target dose was found to be 2,673 μSv/Gy corresponding to a total dose of 120 mSv. For organs in the chest region such as the lungs and thymus, average organ doses of 18 and 32 mSv were calculated for the total target dose, respectively. The breast dose was 17 mSv, while the heart dose was 8.3 mSv. For the liver and stomach, the obtained average doses were 4.1 and 3.4 mSv, respectively. Gonad doses were 1.1 and 0.6 mSv for the ovaries and testes, respectively.

## 4 Discussion

TOPAS was chosen in this study, as it is capable of calculating the dose distribution, including the aura consisting of neutrons and gammas (25, 35). Moreover, it was shown that TOPAS is able to simulate multiple Coulomb scattering in the lucite RS used in the present work with sufficient accuracy (26). As a first verification step of the MC framework, the in-field dose distribution was compared to RayStation, showing an excellent agreement in the 3D global gamma test as shown in **Figure 3**. However, the more sensitive local 3D gamma test revealed discrepancies outside the volume, which, as described in the work of (25), are due to the fact that RayStation simplifies the transport of secondary particles except for protons. For this reason, TOPAS was used to determine the out-of-field doses. Detailed modeling of the beam was performed, as well as detailed modeling of the geometry of the treatment room, including the gantry pit, to account for all possible sources of secondary particles resulting from interactions between particles scattered and generated in the phantom, as well as the environment (33, 34).

A good agreement was observed between TLD measurements, MTS-7 type measuring the non-neutron component of the mixed radiation field, and TOPAS simulations tallying the proton and gamma component. A slight overestimation by MTS-7 detectors was however observed, which could be due to the slightly increased response of MTS detectors for protons (11). We assigned an uncertainty to this potential error of 5% (*k* = 1) in TLD data. Nevertheless, we did not use the spectra as input and assumed a uniform distribution of proton energies, which could be off for certain locations in the field, particularly when protons reach the end of their range. Nevertheless, we did not correct MTS-7 data for non-linearity in the energy response for protons and photons. Even though this can be considered a limitation, we do believe that the impact will be very small, and we considered it in the uncertainty of the detector data. Moreover, the mentioned 12% overresponse of LiF-based TLDs for out-of-field measurements in photon beams does not apply to PT, as out-of-field spectra are much softer than in proton beams (36, 37). Furthermore, it should be noted that in the three outmost TLD positions, there is an underestimation of the dose with TLDs, which is most likely due to the very low doses measured in these positions and uncertainties associated with the background signal (11%).

Another reason for the slight overestimation of TLDs could be the contribution from heavy charged particles or fragments created by secondary neutrons in the phantom itself. This was not considered in the simulation due to the very low statistics of such particles but could have resulted in a signal in MTS-7 detectors. Still, heavy charged particles generated outside the TLD volume and created in the CIRS phantom could contribute, but due to their short range, the likelihood of reaching the TLD detector is small. Moreover, the sensitivity of TLD detectors for heavy charged particles is low, as the TL efficiency depends largely on the ionization density, as studied experimentally and using microdosimetric models (11, 38).

The use of Li-6-enriched LiF : Mg,Cu,P (MCP-6) detectors allowed us to assess the thermal neutron’s contribution due to the high ^6^Li(n, *α*)^3^H cross section for thermal neutrons. However, it should be noted that the quantification of thermal neutrons and the use of appropriate conversion factors are subject to large uncertainties as previously described (8). The method assumes that the dose is deposited only by thermal neutrons, and neutrons are isotropic. Moreover, corrections are applied for superficial dose deposition in TLDs and, therefore, a decreased light attenuation. No information about the energy spectrum and angular distribution was obtained, and therefore, the calculated conversion factor has a large uncertainty (100%). In the current study, the experimental results are always higher as compared to the simulated data, which were the highest close to the field where a 15-fold higher dose was measured. This large discrepancy can be explained not only by the large measurement uncertainty but also by uncertainties in the TOPAS simulations related to the detailed modeling of beam, gantry, bed, walls, and other room components. As thermal neutrons are created by neutrons slowing down during collisions, it is very challenging to model these accurately. However, when moving out-of-field, the experimental data were a factor of 2 higher than simulated data, which could be explained by the more isotropic nature of the thermal neutrons when moving further away from the isocenter. Still, it was observed that the contribution of thermal neutrons to the total neutron dose equivalent is very small (within 1%). Similar findings, with thermal neutron doses contributing <% to the total neutron dose equivalent, have also been observed in (39).

In general, the agreement between the measured and simulated neutron doses was good, despite the large uncertainties of the measurements performed with bubble detectors (20%, *k* = 1). Moreover, this uncertainty did not consider potential dependence on their response as a function of neutron energies. In fact, their response is well characterized until 20 MeV, above which response is not fully described. Nevertheless, previous data using BD-PNDs in PT have shown good agreement with *H*_p_ (10) reference measurements (39). Still, an underestimation was measured in some positions, which could be assigned to the use of PMMA slabs instead of the anthropomorphic materials containing tissue materials. The discrepancy is the largest, and up to 50% lower for measured data compared to simulated data, in the area where there is lung material. As the TOPAS model was based on the phantom’s CT and did not model the PMMA slabs and the exact material densities during measurements, this could be the cause of the discrepancy. Although this could be considered a limitation of the study, the dose data, namely, tissue dose, as determined in the TOPAS simulations are highly relevant for translation to the clinic and epidemiology. Another reason for the discrepancy could come from the uncertainty in neutron dose simulations due to missing cross-section data above 20 MeV for which many codes need to rely on the use of nuclear models. Several models are available; however, it is still open which models are more suitable. The choice of the binary intra-nuclear cascade (BIC) model in GEANT4 within this study was based on previous data demonstrating a good agreement with experiments (25, 34, 40).

Once validated, the MC simulation framework allowed to calculate the total dose, which was assessed as the dose quantity *dose equivalent*, considering the biological effectiveness of the radiation, which is dependent on the radiation type and energy. In previous studies, usually, the total dose in out-of-field positions is calculated considering only the neutron and gamma dose, for example, in (12, 14). Our study, however, demonstrates that closer than 30 cm from the isocenter, the contribution from primary protons is significant. We therefore added this contribution by applying a generic RBE of 1.1 for protons. We did not calculate the proton’s energy, and we do recognize that this calculation may be a simplification, as the RBE will depend on the proton’s linear energy transfer (LET). Still, the RBE-LET relationship is under investigation and would require more extensive calculations of (micro)dosimetric quantities, which we considered out of the scope of this paper.

Unfortunately, the calculation of the total dose equivalent was not possible from the experimental detectors, as TLDs will measure, apart from the gamma dose, both the primary and secondary protons. When summing these to the neutron doses from BD-PNDs, this will lead to an overestimation due to the double counting of recoil protons. Research is ongoing on how to combine different detector systems, with various response functions, to overcome the challenges of mixed radiation fields and to allow for an accurate experimental measurement of the total dose equivalent in the future. Similar issues are encountered for dosimetry in space where also complex mixed radiation fields consisting of neutrons, photons, protons, and heavier ions exist (41). In space, often silicon telescopes and other spectroscopic devices are used in order to be able to separate the different radiation field components and to obtain an estimation of the total dose equivalent. However, such detectors are too bulky to be used in phantom measurements in PT.

Previous works mainly evaluated out-of-field doses in PT through experimental measurements in water phantoms (1) or anthropomorphic phantoms (42–44). Only few studies have modeled the PT beam in detail to allow MC calculations of out-of-field doses in PT (45–47). Studies connecting MC and experimental data in PT are mostly limited to measurements with ambient monitors or Bonner sphere systems in the room (33, 34, 48). Such studies are lacking in phantom measurements, which are of utmost importance for patient care. Our study provides a first step in the development of an MC framework that allows us to fully characterize the out-of-field radiation field and eventually could lead to tools for dose and risk optimization in children.

It is important to make the framework less computationally demanding, as now a very detailed beam model is used. In the same way as it was done in the study from (34), it will be important to identify the origin of the secondary radiation in the beamline component and to allow simplification of the beam model. Therefore, in the next developments, it must be considered which components of the simulation the complexity can be reduced without changing the simulation results to such an extent that it no longer coincides with the present result within the standard statistical uncertainty. For this purpose, a traceable approach is to determine which part of the treatment room has the largest share in the secondary particle generation or scattering. In this way, the dimensions of the room can presumably be reduced, which is synonymous with a reduction in the simulation time. Likewise, the origin of the secondary radiation depending on the direction of flight and momentum of the protons escaping the nozzle, as well as the scattering in the body and phantom, needs to be analyzed with the scope of simplifying the applied irradiation field, which in turn reduces the effort required to generate the phase space files and ultimately eliminates the time-consuming step function feature with the equally time-consuming phase space sampling of each spot.

The closest comparison of our experimental data to literature could be made to two studies performed within EURADOS WG9 (42, 43), measuring out-of-field doses during PT in the same anthropomorphic 5-year-old phantom treated for a brain tumor (6-cm diameter). One study described the response of passive detector systems in PT out-of-field dosimetry (42), where no RS (no RS) was used, while another study verified the impact of using an RS or 3D-printed beam compensator (BC) on the out-of-field doses (43). At 12 cm, the neutron dose equivalent data were 120 μSv/Gy (no RS) in the study from (42) versus 130 μSv/Gy (BC) and 180 μSv/Gy (RS) in the study from (43). Our data reported a dose of 260 μSv/Gy, which could be due to the larger volume in the current study (195.2 cm^3^) compared to the previous studies (65 cm^3^) (42, 43). Interestingly, these studies also compared data to photon plans for the same phantom, tumor size, and location, revealing that intensity-modulated radiation therapy (IMRT) and 3D conformal radiation therapy (3D-CRT) (49) are at least one order of magnitude higher than PT at 30 cm. As part of another paper within this special issue, the same case was treated with IMRT and volumetric modulated arc therapy (VMAT), and we noted a reduction in out-of-field dose of a factor of approximately 5, close to the field for both IMRT and VMAT, while at 30 cm, the difference was a factor of 35 and 20 lower for PT as compared to IMRT and VMAT, respectively.

We reported on organ doses for a target dose of 50.4 Gy(RBE) by multiplying the normalized doses with the physical target dose (45.8 Gy). Nevertheless, in the clinical treatment course, a second beam set delivered 3.6 Gy(RBE) in two fractions to a PTV, which was cut at the inferior side to protect the spinal cord. The corresponding reduction in the volume of the modified PTV amounted to 2.6%. Thus, the out-of-field contributions of the second beam set can be approximated by the corresponding values of the initial beam set.

Looking into the organ doses, the thyroid dose was the highest, yielding 120 mSv, while other organ doses ranged between 18 mSv for the lungs and 0.6 mSv for the testes. According to BEIR VII (50), the lifetime attributable risk (LAR) for cancer incidence and for an exposure at 5 years old is most elevated for the breast, lungs, and thyroid with LAR values of 914, 608, and 419/10^5^/0.1 Gy for women, respectively, while for men, the values were 261 and 76/10^5^/0.1 Gy for the lung and thyroid, respectively. Applying these risk factors to our data, we estimated a risk for secondary thyroid cancer of 0.6% for women and 0.1% for men. The risk for breast cancer was 0.2%. However, we should be aware that these risk models are mainly for low doses and low dose rates, and they cannot easily be extrapolated to radiotherapy where the dose is fractionated and organ dose is heterogeneous. These estimations should be considered with even more caution in the context of PT, as the effects of high-LET particles (i.e., protons and heavier ions) are outside the scope of the BEIR VII report.

Knowledge about potential long-term sequelae of treatment modalities needs precise data on the oncologic treatment, related to not only radiotherapies, such as the dose-volume histogram for every OAR (in the field and out of the field), but also the cumulative dose of every drug of chemotherapy (including new molecules and corticosteroids) and precise information on surgeries. This information needs to be complemented with a long period of follow-up.

The HARMONIC project was set up to provide direct evidence of the late health effects of low, moderate, and high radiation doses from modern radiotherapy techniques using protons or photons. Following up pediatric patients treated with PT will strengthen the epidemiological basis for assessing radiation risk in pediatric patients and will provide complementary information to the contribution from the large historical childhood survivor cohorts treated prior to 2000, which did not include new treatment modalities (51). The HARMONIC project therefore builds the infrastructure and instruments to evaluate the potential health, QoL, and social impacts of medical exposures to ionizing radiation in children, with potential for advanced patient-specific dose reconstruction, as presented here, and mechanistic investigations. It aims at providing evidence on the magnitude of possible cancer and non-cancer effects (including neurovascular, cardiovascular, and endocrine system effects), which may arise following cancer treatment with modern techniques, including PT in pediatrics.

## 5 Conclusion

As the role of proton beam therapy is continuously increasing, particularly when very young children are concerned, the understanding of out-of-field doses and their impact on secondary cancer induction is essential. Since the HARMONIC project aims to investigate the incidence of secondary cancer, a reliable calculation of the out-of-field dose is of crucial importance. In this framework, the development of a validated MC system forms an important aspect in the assessment and characterization of out-of-field doses in PT. Once validated, MC simulations allow to fully describe the out-of-field radiation, permitting calculations of appropriate dosimetric quantities needed for the assessment of radiation damage and risks. In this study, the coupling of MC to advanced measurements with different detector types enabled the performance of a proper benchmarking of a widely used MC code, for use in out-of-field dosimetry.

The proposed computational method for calculation of the out-of-the-field dose in PT produces results that are compatible with the experimental data. The validated framework allowed a detailed characterization of the radiation field and the calculation of out-of-field organ doses during PT. The development of such an MC framework could lead to tools for dose and risk optimization in children.

## Data Availability Statement

The raw data supporting the conclusions of this article will be made available by the authors, without undue reservation.

## Author Contributions

MDS-H: experimental design, experimental setup, data analysis, and writing. NV: Monte Carlo simulation design, setup and execution, experimental setup, data analysis, and writing. CB: treatment planning, experimental setup, and editing. JE: Monte Carlo geometry coding. JW: preparation of Monte Carlo configuration files and data analysis. RN: experimental setup. OVH: experimental analysis. JD: Monte Carlo simulations. FS: Monte Carlo data analysis and experimental setup. FV: simulation validation, data analysis, and writing—review. SR: analytical model and writing—review. GB: experimental setup writing—review. US: simulation validation and writing—review. MR: Monte Carlo data analysis. BT: clinical analysis, writing—review, and methodology. IT-C: epidemiological review, writing—review, and methodology. LB: conceptualization, supervision, writing, and writing—review. All authors contributed to the article and approved the submitted version.

## Funding

The presented research has been funded by the HARMONIC project. The HARMONIC project (Health effects of cArdiac fluoRoscopy and MOderN radIotherapy in paediatriCs) has received funding from the Euratom research and training program 2014-2018 under grant agreement No 847707. MR acknowledges funding from the Sistema Nacional de Investigación de Panamá. IT-C acknowledges support from the Spanish Ministry of Science and Innovation and State Research Agency through the “Centro de Excelencia Severo Ochoa 2019-2023” Program (CEX2018-000806-S) and support from the Generalitat de Catalunya through the CERCA Program.

## Conflict of Interest

The authors declare that the research was conducted in the absence of any commercial or financial relationships that could be construed as a potential conflict of interest.

## Publisher’s Note

All claims expressed in this article are solely those of the authors and do not necessarily represent those of their affiliated organizations, or those of the publisher, the editors and the reviewers. Any product that may be evaluated in this article, or claim that may be made by its manufacturer, is not guaranteed or endorsed by the publisher.
